# Nanocomposites of Graphene Oxide—Silver Nanoparticles for Enhanced Antibacterial Activity: Mechanism of Action and Medical Textiles Coating

**DOI:** 10.3390/ma15093122

**Published:** 2022-04-26

**Authors:** Agata Lange, Ewa Sawosz, Mateusz Wierzbicki, Marta Kutwin, Karolina Daniluk, Barbara Strojny, Agnieszka Ostrowska, Barbara Wójcik, Maciej Łojkowski, Marcin Gołębiewski, André Chwalibog, Sławomir Jaworski

**Affiliations:** 1 Department of Nanobiotechnology, Institute of Biology, Warsaw University of Life Sciences, Ciszewskiego 8 Str., 02-786 Warsaw, Poland; agata_lange@sggw.edu.pl (A.L.); ewa_sawosz_chwalibog@sggw.edu.pl (E.S.); mateusz_wierzbicki@sggw.edu.pl (M.W.); marta_prasek@sggw.edu.pl (M.K.); karolina_daniluk@sggw.edu.pl (K.D.); barbara_strojny@sggw.edu.pl (B.S.); agnieszka_ostrowska@sggw.edu.pl (A.O.); barbara_wojcik@sggw.edu.pl (B.W.); 2 Faculty of Materials Science and Engineering, Warsaw University of Technology, Wołoska 141 Str., 02-507 Warsaw, Poland; 00183042@pw.edu.pl; 3 Centre for Advanced Materials and Technology CEZAMAT, Warsaw University of Technology, Poleczki 19 Str., 02-822 Warsaw, Poland; 4 Department of Animal Breeding, Institute of Animal Sciences, Warsaw University of Life Sciences, Ciszewskiego 8 Str., 02-786 Warsaw, Poland; marcin_golebiewski@sggw.edu.pl; 5 Department of Veterinary and Animal Sciences, University of Copenhagen, Groennegaardsvej 3 Str., 1870 Frederiksberg, Denmark; ach@sund.ku.dk

**Keywords:** nanocomposite, silver nanoparticles, graphene oxide, antibacterial, coating

## Abstract

The resistance of microorganisms to antibiotics is a crucial problem for which the application of nanomaterials is among a growing number of solutions. The aim of the study was to create a nanocomposite (composed of graphene oxide and silver nanoparticles) with a precise mode of antibacterial action: what enables textiles to be coated in order to exhibit antibacterial properties. A characterization of nanomaterials (silver nanoparticles and graphene oxide) by size distribution, zeta potential measurements, TEM visualization and FT-IR was performed. The biological studies of the nanocomposite and its components included the toxicity effect toward two pathogenic bacteria species, namely *Pseudomonas aeruginosa* and *Staphylococcus aureus*, interaction of nanomaterials with the outer layer of microorganisms, and the generation of reactive oxygen species and lipid peroxidation. Afterwards, antibacterial studies of the nanocomposite’s coated textiles (cotton, interlining fabric, polypropylene and silk) as well as studies of the general toxicity towards a chicken embryo chorioallantoic membrane model were conducted. The toxicity of the nanocomposite used was higher than its components applied separately (zones of growth inhibition for *P. aeruginosa* for the final selected concentrations were as follows: silver nanoparticles 21 ± 0.7 mm, graphene oxide 14 ± 1.9 mm and nanocomposite 23 ± 1.6 mm; and for *S. aureus* were: silver nanoparticles 27 ± 3.8 mm, graphene oxide 14 ± 2.1 mm, and nanocomposite 28 ± 0.4 mm. The viability of *P. aeruginosa* and *S. aureus* after treatment with selected GO-Ag decreased to 27% and 31%, respectively, compared to AgNPs, when the viability of both species was 31% and 34%, accordingly). The coated textiles showed encouraging antibacterial features without general toxicity towards the chicken embryo chorioallantoic membrane model. We demonstrated that graphene oxide might constitute a functional platform for silver nanoparticles, improving the antibacterial properties of bare silver. Due to the application of the nanocomposite, the textiles showed promising antibacterial features with a low general toxicity, thereby creating a wide possibility for them to be used in practice.

## 1. Introduction

The introduction of antibiotics into medicine has revolutionized the therapy of infectious diseases. Antibiotics are used primarily to fight infections and as growth and health promoters in livestock [1]. However, the unjustified use and overuse of antibiotics has contributed to an increase in the number of resistant bacteria strains [2]. Among Gram-positive bacteria, methicillin-resistant *Staphylococcus aureus* and vancomycin-resistant *Enterococcus* sp. cause great difficulties in treatment. The most serious Gram-negative infections are caused by *Enterobacteriaceae*, *Pseudomonas aeruginosa* and *Acinetobacter* [3]. Every time a new antimicrobial is introduced, resistant strains emerge. Infections caused by antibiotic-resistant germs are difficult and sometimes impossible to treat. Growing antibiotic resistance is forcing the search for new, alternative antibacterial agents. Nanoparticles (NPs) are often used to target microorganisms as an alternative to antibiotics. Furthermore, nanomaterials may be used effectively in biomedical practice to coat textiles, which is what ensures them antibacterial properties [4]. Among popular textiles, especially important are medical types which are popular in everyday life (for example those from which face masks are produced) and, due to the coating, do not contain bacteria, especially drug-resistant ones. 

Most of the mechanisms of antibiotic resistance are not related to nanoparticles nor nanomaterials. The toxicity of nanoparticles is based on the mechanical damage of the cell wall and membrane, ion secretion and the activation of oxidative stress [5]. Studies have shown that many NPs have antibacterial activity, including silver nanoparticles (AgNPs), gold, zinc, copper and iron [6,7,8,9,10]. However, most research has focused on the use of AgNPs. It has been demonstrated that the bactericidal properties of AgNPs are strongly influenced by their size, shape and concentration [11,12]. Various shapes of nanoparticles show different antibacterial properties. Pal et al. [13] showed that triangular-shaped nanoparticles exhibit stronger antibacterial properties than rod-shaped and spherical nanoparticles. Different surface chemistry and functionalization may change the interaction of nanoparticles with bacteria and inhibit their antibacterial activity. Some studies have shown that smaller diameter nanoparticles have stronger antibacterial properties [14]. The smaller size also promotes the secretion of ions that destroy bacterial cell structures. Silver nanoparticles can accumulate on the bacterial cell membrane and cause denaturation of membrane proteins. They can also penetrate the bacterial cell, damaging the organelles [6]. However, silver nanoparticles often agglomerate upon contact with bacteria. The inhibitory effect of aggregated nanoparticles decreases with an increase in the degree of aggregation of AgNPs [15]. To reduce this problem, it is possible to synthesize nanocomposites containing AgNPs, ensuring a better and more stable dispersion of the AgNPs. As we showed in a previous study [16], one of the materials that can act as a carrier for AgNPs is graphene oxide (GO). It is water-soluble and provides a large platform for convenient functionalization-based molecule attachment [17]. Nanocomposites can provide a better dispersion of nanoparticles and reduce their agglomeration. Factors such as size and agglomeration are especially important for nanocomposites, since, depending on these, some nanomaterials may not be able to penetrate the bacteria cell wall [18]. However, an increase in the exposed surface while creating platforms for AgNPs also causes a prolonged release of active Ag ions which damage the bacteria’s external membrane by direct contact [19].

In this study, we hypothesized that GO-based nanocomplexes (GO-Ag) will have a stronger antibacterial effect than bare AgNPs. Nanoparticles distributed evenly on the surface of graphene may have a stronger contact with the surface of bacterial cells, leading to their damage. The objective of this study was to evaluate the antimicrobial activity of GO decorated with AgNPs compared to bare AgNPs with the distinction of a nanocomposite mechanism, using the bacteria *Pseudomonas aeruginosa* and *Staphylococcus aureus.* Thereafter, its antibacterial features on medical textiles were assessed. 

In order to examine the initial assumptions, the following analyses were carried out: physicochemical characterization of nanomaterials, evaluation of bacterial growth by the agar well diffusion method, ultrastructural analysis of bacteria cells by transmission electron microscopy (TEM), determination of bacteria viability by XTT assay, evaluation of reactive oxygen species (ROS) generation, assessment of lipid peroxidation by MDA assay and estimation of antibacterial activity of textile materials coated with nanomaterials by the ISO 20645:2004 standard, as well as general toxicity by chorioallantoic membrane (CAM) assay. 

## 2. Materials and Methods

### 2.1. Bacterial Culture 

Microbial strains (*P. aeruginosa* (ATCC 27853) and *S. aureus* (ATCC 25923)) were obtained from LGC Standards (Lomianki, Poland). Both strains in the form of spore suspensions were maintained frozen in 20% (*v*/*v*) glycerol at −20 °C. In order to use them in experiments, they were defrosted and purified from glycerol by washing with distilled water. Afterwards, bacterial strains were cultured in Mueller–Hinton broth medium (BioMaxima, Lublin, Poland) and incubated in a shaking incubator at 37 °C overnight. 

Before the experiments, the bacterial cells were adjusted to a dedicated concentration by dilution in a sterile distilled saline solution, based on the McFarland scale [20]. 

### 2.2. Characterization of Nanoparticles

Silver nanoparticles were obtained from Nano-Tech (Warsaw, Poland) and GO was obtained from Advanced Graphene Products (Zielona Gora, Poland). Suspensions of AgNPs (25 μg/mL), GO (5 μg/mL) and their mixture GO-Ag (AgNPs (25 μg/mL) + (GO 5 μg/mL)) were prepared in deionized water and sonicated for 30 min before usage in each experiment. In order to specify the physicochemical properties of the nanocomposites, the individual characteristics of the AgNPs and GO as well as their combination were defined with the methods previously described in the research of Jaworski et al. [16]. The shape was determined using TEM JEM-1220 (JEOL, Tokyo, Japan) at 80 keV. Size distribution (dynamic light scattering method) and zeta potential (laser Doppler electrophoresis method) measurements were carried out using the Zetasizer Nano-ZS ZEN 3600 (Malvern Instruments Ltd., Malvern, UK) at room temperature (23 °C). 

FT-IR measurements were performed using a Nicolet iS10 spectrometer (Thermo Fisher Scientific, Waltham, MA, USA): 200 mg of KBr powder (Sigma-Aldrich, Munich, Germany) was pressed together under 10 Atm to form discs, and then a droplet of the suspension (concentration of 75 μg/mL) was pipetted onto the KBr disc and dried in a vacuum overnight. The dried KBr with the analyte was then once again milled and dried. The operation was repeated multiple times if necessary. The discs were investigated in transmittance mode. 

### 2.3. Agar Well Diffusion Method 

The well diffusion test was performed in order to determine the antibacterial activity of the nanoparticles. Different concentrations of nanoparticles were tested in order to select the optimal antibacterial concentration of the nanocomposites created (AgNPs-50, 25, 10, 5, 2.5 (μg/mL); GO-10, 5 (μg/mL); GO-Ag-Ag 25 (μg/mL) + GO 5 (μg/mL); Ag 25 (μg/mL) + GO 10 (μg/mL)). Nanoparticles were prepared by dilution in deionized water and sonicated for 30 min before usage. The bacterial inoculum was prepared by adjusting the turbidity of the suspensions to match 0.5 McFarland standard, which is equivalent to 1.5 × 10^8^ cells/mL [20]. A total of 100 μL of both strains of microorganisms was spread over the surface of a Mueller–Hinton agar plate (BioMaxima, Lublin, Poland). Wells (made by a 13 mm diameter sterile cork borer) were punched in culture agar plates, and 100 μL of nanoparticles as the substances tested and 100 μL of distilled water as the control were placed into each well. The plates were incubated at 37 °C for 24 h. The area of inhibition was identified as a clear zone around a well and the zone was measured in mm.

### 2.4. Ultrastructural Analysis by Transmission Electron Microscopy (TEM)

The effect of the interaction between the nanoparticles used and the bacteria cells was evaluated by transmission electron microscope JEM-1220 (JEOL, Tokyo, Japan), operated at a voltage of 80 keV. The overnight bacteria suspension was adjusted to 1.5 × 10^8^ cells/mL and treated with AgNPs, GO and GO-Ag. Droplets of each sample were placed onto TEM grids (Formvar on 3 mm 200 Mesh Cu Grids, Agar Scientific, Stansted, UK), and the samples were observed immediately. 

### 2.5. Viability XTT Assay

The viability rate was determined by Cell Proliferation Kit II (Cat. No. 11465015001, Merck, Darmstadt, Germany), where the tetrazolium salt XTT (sodium 3′-[1-[(phenylamino)-carbony]-3,4-tetrazolium]-bis(4-methoxy-6-nitro) benzene-sulfonic acid hydrate) is reduced by dehydrogenase enzymes in metabolically active cells, giving a colored formazan product which is measured spectrophotometrically. Bacterial strains were cultured in Mueller–Hinton broth medium (BioMaxima, Lublin, Poland) in a shaking incubator at 37 °C overnight. Nanoparticles were prepared by dilution in deionized water and sonicated for 30 min before usage. The final concentrations of the nanoparticles were as follows: AgNPs (0.8; 1.56; 3.125; 6.25; 12.5; 25 (μg/mL)), GO (5 μg/mL) and GO-Ag (Ag 0.8 + GO 5; Ag 1.56 + GO 5; Ag 3.125 + GO5; Ag 6.25 + GO5; Ag 12.5 + GO 5; Ag 25 + GO 5 (μg/mL)). A total of 90 μL of bacterial suspension (5 × 10^5^ cells/well) was placed in a 96-well plate and treated with 10 μL Ag, GO and GO-Ag nanoparticles for 24 h in a bacteriological incubator under standard conditions (37 °C). Subsequently, 50 μL of XTT mix was added into each well and incubated for 3 h at 37 °C. Absorbance at 450 nm was measured using a microplate Elisa reader (Infinite M200, Tecan, Durham, NC, USA). The results were repeated a minimum of three times for each group. Cell viability was expressed as a percentage of the optical density of the test sample reduced by a blank probe in relation to the optical density of the control reduced by a blank probe, where the control is the optical density of the bacterial suspension without nanoparticles, and the blank probe is optical density of the wells without bacterial cells. 

### 2.6. ROS Production

The detection of intracellular ROS was determined using the Fluorometric Intracellular Ros Kit (Cat. No. MAK143, Sigma, St Louis, MO, USA), with the main principle being that ROS reacts with a sensor in the cytoplasm, giving a fluorometric product. Bacterial strains were cultured in Mueller–Hinton broth medium (BioMaxima, Lublin, Poland) in a shaking incubator at 37 °C overnight. Nanoparticles were prepared by dilution in deionized water (final concentration used: AgNPs 25 μg/mL, GO 5 μg/mL and GO-Ag (Ag 25 μg/mL + GO 5 μg/mL)) and sonicated for 30 min before usage. Bacterial inocula (5 × 10^5^ cells per well) with test nanoparticles (final volume 100 μL) were placed in 96-well plates and incubated at 37 °C for 24 h. A Master Reaction Mix was prepared according to protocol and 50 μL of the mixture was added to each well. Shortly after, the fluorescence measurements were conducted with an excitation wavelength at 490 nm and an emission wavelength at 525 nm using the microplate reader Infinite M200 (Infinite M200, Tecan, Durham, NC, USA). The results were replicated a minimum of three times for each group.

### 2.7. Lipid Peroxidation (MDA) Assay

Lipid peroxidation was determined by MDA assay kit (Cat. No. MAK085, Sigma, St Louis, MO, USA) in which malondialdehyde (MDA), as a by-product of lipid peroxidation of the bacterial cell membrane, reacts with thiobarbituric acid (TBA), giving the fluorimetric product proportional to the MDA present. The bacterial strains were cultured in Mueller–Hinton broth medium (BioMaxima, Lublin, Poland) in a shaking incubator at 37 °C overnight. The nanoparticles were prepared by dilution in deionized water (final concentration used: AgNPs 25 μg/mL, GO 5 μg/mL and GO-Ag (Ag 25 μg/mL + GO 5 μg/mL)) and sonicated for 30 min before usage. After being exposed to the nanoparticles for 24 h, bacterial inoculum (1 × 10^6^) were homogenized by MDA Lysis Buffer and centrifuged at 13,000× *g* for 10 min, according to the protocol. A total of 200 μL of supernatant was placed into a microcentrifuge tube, and then 600 μL TBA was added and incubated at 95 °C for 60 min. A total of 200 μL of the mixture was placed into a 96-well plate and the absorbance at 532 nm was measured. The test was replicated a minimum of three times for each group.

### 2.8. Textile Fabrics–Determination of Antibacterial Activity–Agar Diffusion Plate Test (ISO 20645:2004)

The antibacterial activity of the textile fabrics was determined according to normative test ISO 20645:2004. Four types of medical materials (silk, polypropylene, cotton and interlining fabric) in round form (diameter 25 mm) were covered with 500 μL of the GO-Ag nanocomposite by ultrasonic treatment for 30 min.

The agar phase consisted of two distinct layers. The lower layer was agar only and the upper layer was agar with a bacteria culture. First, 10 mL of nutrient agar (BioMaxima, Lublin, Poland) was placed on Petri dishes (90 mm in diameter). Then, 150 mL of cooled nutrient agar was mixed with 1 mL of bacterial suspension (1.5 × 10^8^ cells/mL) and poured on the surfaces of prepared Petri dishes. After complete solidification, textile fabrics were placed on the surface of the inoculum medium, ensuring that they adhered evenly to the surface. The plates were incubated for 24 h at 37 °C and the results were determined by the presence or absence of bacterial growth in the area of contact between the sample and the agar.

### 2.9. Chorioallantoic Membrane (CAM) Assay

The textile fabric implants were made of four types of medical materials (silk, polypropylene, cotton and interlining fabric). Implants of 10 mm diameter were covered with 200 μL of the GO-Ag nanocomposite by ultrasonic treatment for 30 min to complete surface coverage.

Fertilized eggs (line Ross 308) were obtained from a local hatchery (Marylka, Mazovian voivodship, Poland). The eggs were cleaned and sterilized with UVC and then kept in standard conditions (temperature 37 °C, humidity 60%, turned once per hour) until the sixth day of chicken embryo development. Then, small holes were made in the shell above the air space and the textile fabric implants (10 mm diameter) were placed on the chicken embryo chorioallantoic membrane. The eggs were incubated in standard conditions to day seven of chicken embryo development. Thereafter, implants with CAM were affixed with 1.5 mL 4% paraformaldehyde and incubated for 30 min at 4 °C. Then, the implants with CAM were slightly cut out using a sterile scalpel and observed under a stereomicroscope (SZX10, Olympus Corporation, CellD software version 3.1, Tokyo, Japan). Angiogenesis was measured by the density and length of the blood vessels that formed on the implant surface using ImageJ software version 1.50e with a plugin Vessel analyzer. All measurements were repeated a minimum of three times.

### 2.10. Statistics

In this study, all data are represented as mean ± standard deviation. For statistical analysis, one-way analysis of variance with the post-hoc Tukey test (HSD) was performed using GraphPad Prism 9 software, and the significance level was considered at *p*-value ≤ 0.05.

## 3. Results

### 3.1. Characterization of Nanoparticles

**Figure 1:**
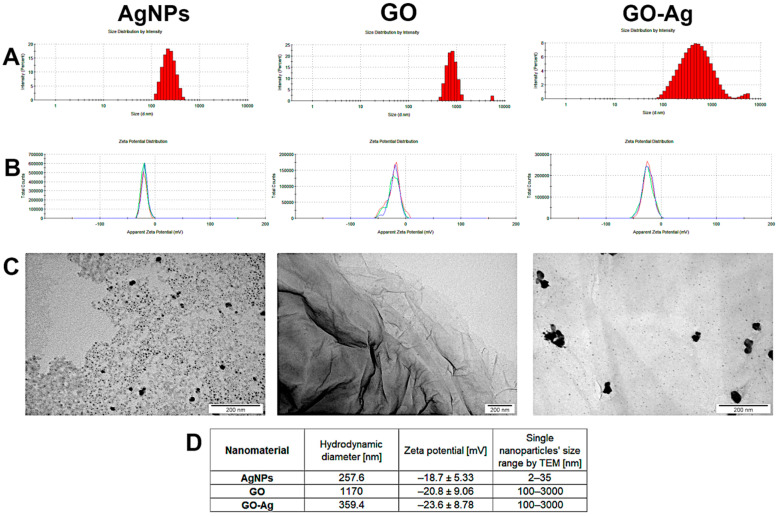
Physicochemical characterization of nanomaterials-silver (AgNPs), graphene oxide (GO) and GO with AgNP complexes (GO-Ag): (**A**) hydrodynamic diameter measured by dynamic light scattering; (**B**) zeta potential of nanomaterials measured by laser Doppler electrophoresis, three colors (blue, green, red) represent repeated measurements of the same sample; (**C**) TEM visualization; (**D**) values of measurements. Each measurement was repeated three times.

The physicochemical characteristic of nanomaterials allows the interpretation of their properties in relation to living cells, tissues and organisms. The results from the size distribution, zeta potential and visualization of nanoparticles/agglomerates are presented in Figure 1. The hydrodynamic diameter of the AgNPs exceeded 200 nm, but the slightly negative zeta potential and visualization by TEM indicated that they create agglomerates. In the case of GO, the large hydrodynamic diameter (1170 nm) is related to the GO’s shape; its visualization by TEM revealed it to be in the form of flakes, not a sphere. Finally, complexes created between GO and AgNPs were visualized, showing a variable zeta potential as well as differentiated size; however, the value of the zeta potential was the most negative, approaching the limit value of ±30 mV (zeta potential value for GO-Ag was—23.6 ± 8.78 mV).

**Figure 2:**
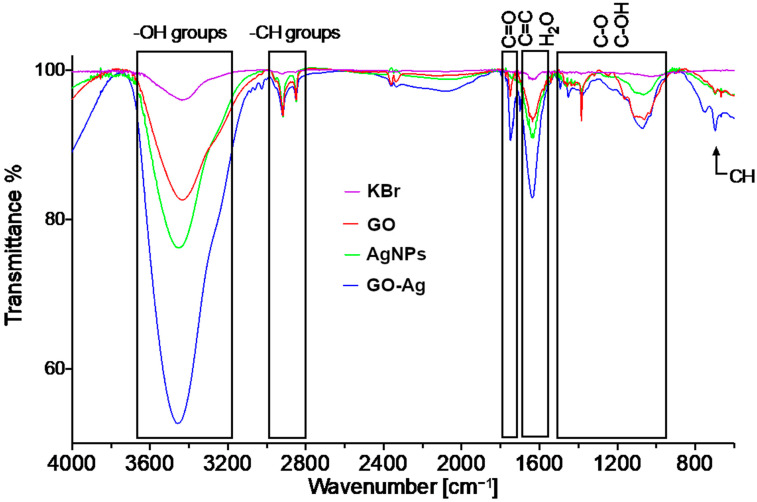
FT-IR transmittance spectrum of graphene oxide (GO), silver (AgNPs) and GO with AgNP complexes (GO-Ag) in comparison to KBr spectrum.

The FTIR spectra of the pellets prepared from KBr and the analytes are presented in Figure 2. Wide bands at 3430 cm^−1^ related to the -OH groups were found in all spectra. The bands at 2913 cm^−1^ and 2843 cm^−1^ are related to the CH groups. The band at 1743 cm^−1^ in the GO spectrum is related to the carboxyl C=O group. The band at 1640 cm^−1^ in the case of GO should be associated with the overlapping H-O-H and C=C vibrations [16]. The bands found at 1445 cm^−1^, 1374 cm^−1^ 1160 cm^−1^ 1066 cm^−1^ and 1015 cm^−1^ are related to the C-O and C-OH hydroxyl groups. The AgNPs spectrum is related to the moieties attached to the surface of the nanoparticles. The bands related to the CH groups are also present at around 2900 cm^−1^ band. The band at around 1640 cm^−1^ is due to the OH scissoring vibrations. The 1374 cm^−1^ and the 1066 cm^−1^ bands can be ascribed to the C-O and the C-OH vibrations as well [21]. The spectra of the GO and AgNPs composites express the composite characteristics of the two spectra.

### 3.2. Agar Well Diffusion Method

**Figure 3:**
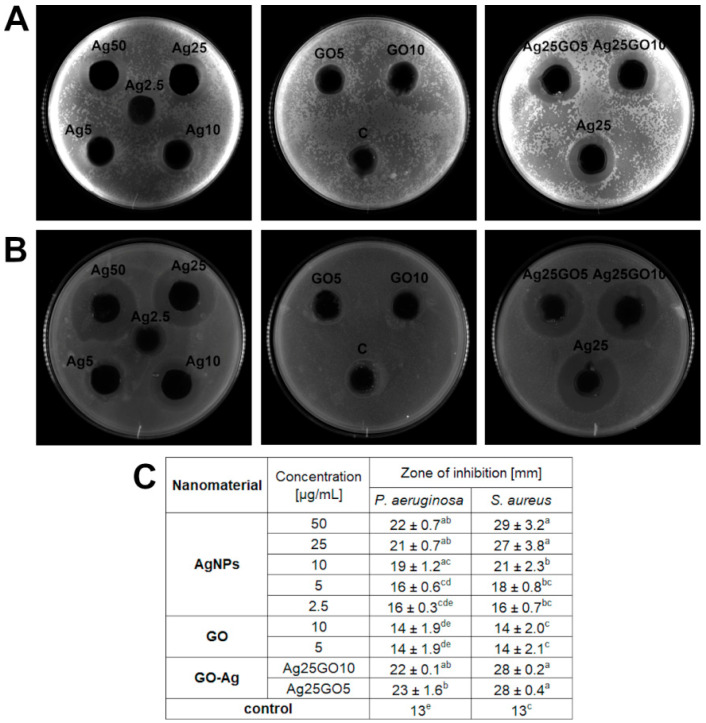
Antibacterial effect of nanomaterials-silver nanoparticles (AgNPs), graphene oxide (GO) and GO with AgNP complexes (GO-Ag) by agar well diffusion method: (**A**) effect on *P. aeruginosa* (sample picture); (**B**) effect on *S. aureus* (sample picture); (**C**) zone of inhibition [mm] after treatment with nanomaterials. Ag25GO5 means the composite consisting of AgNPs at concentration 25 μg/mL and GO at 5 μg/mL; Ag25GO10 means the composite consisting of AgNPs at concentration 25 μg/mL and GO at 10 μg/mL; different letters (a–e) indicate significant differences between groups within bacteria species (*p*-value ≤ 0.05).

We examined the antibacterial effect of AgNPs, GO and GO-Ag towards two bacteria strains—*S. aureus* and *P. aeruginosa* (Figure 3). The GO-Ag nanocomplex caused higher antibacterial activity but the zone of inhibition created by the complex was indistinguishable between the two different concentrations of GO, so that the combination of silver nanoparticles and GO was still the most effective towards bacteria species. Based on our results, it is noticeable that growth inhibition depends on the microbial species. *S. aureus* showed much bigger zones of growth inhibition in all samples of AgNPs and GO-Ag nanocomposites than *P. aeruginosa.* Thus, *S. aureus* was more sensitive than *P. aeruginosa* to exposure to the substances tested.

### 3.3. Ultrastructural Analysis by TEM

**Figure 4:**
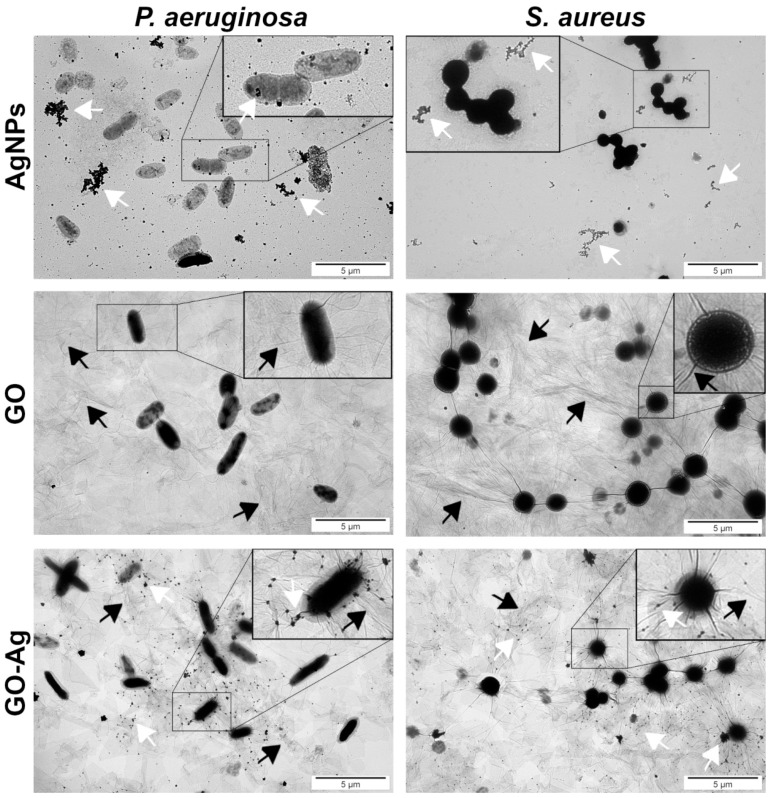
Ultrastructural analysis of bacteria using TEM; silver (AgNPs), graphene oxide (GO) and GO with AgNP complexes (GO-Ag). White arrows point to AgNPs/agglomerates and black arrows point to bending of GO flakes.

One of the most possible mechanisms of interaction of nanomaterials with bacteria cells is the disruption of the cell membrane and wall, which both form an outer cover of bacteria. Bare AgNPs and GO were located near cells (Figure 4). AgNPs were found in direct contact with bacteria; they covered the outer layer of *P. aeruginosa*, but the same location of those nanoparticles was not observed for *S. aureus*. The GO provided a large platform on which bacterial cells were situated. The GO-Ag nanocomplex enabled the AgNPs to wrap the bacteria by using a GO carrier, which ensured the bacteria’s direct exposure to the AgNPs.

### 3.4. Viability XTT Assay

**Figure 5:**
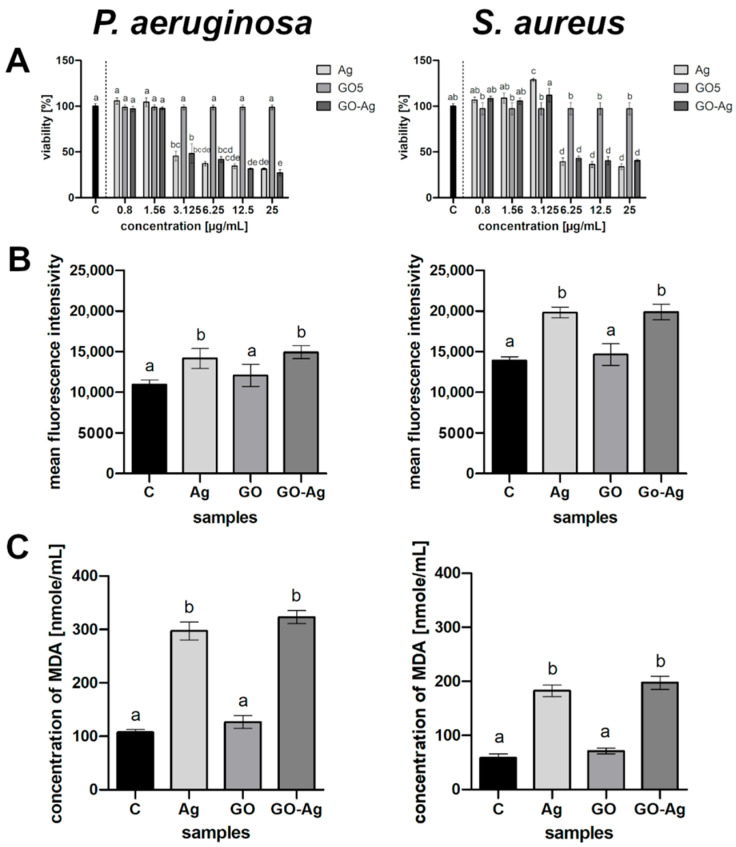
Metabolic activity of bacteria after treatment with silver nanoparticles (Ag), graphene oxide (GO) and GO with AgNP complexes (GO-Ag) in relation to control (C): (**A**) viability determined by XTT analysis, column C is control; (**B**) generation of reactive oxygen species; (**C**) lipid peroxidation determined by MDA assay. The columns are mean values; error bars are standard deviations; different letters (a–e) indicate significant differences between groups within bacteria species (*p*-value ≤ 0.05).

Both AgNPs and GO-Ag affected the bacterial viability in a dose-dependent manner (Figure 5A). The complex decreased the viability of *P. aeruginosa* more than when using the component nanoparticles separately in major probes with diverse AgNP concentrations. However, the same tendency was not observed in the *S. aureus* bacterial culture, although the complex reduced the viability by up to 40%.

### 3.5. ROS Production

In the presented study, all nanomaterials caused the generation of ROS at a higher level compared to the control (Figure 5B). The ROS effect strictly depends on the ROS amount, as it may pose a risk to bacterial function if the optimum is exceeded. The highest ROS production was observed in the GO-Ag complex in both bacterial strains, while the lowest was seen in the GO group. AgNPs led to increased ROS production in all tested microorganisms, though this was only slightly lower than in the complex group.

### 3.6. Lipid Peroxidation (MDA) Assay

Lipid peroxidation was determined to be a result of oxidative stress from the reaction between malondialdehyde (MDA) and thiobarbituric acid (TBA). The highest lipid peroxidation, which was twice as high as the control, occurred after the treatment of the bacteria suspension with GO-Ag within all samples (Figure 5C). The other individual nanoparticles also increased the MDA level; however, GO was the lowest in relation to the control.

### 3.7. Textile Fabrics—Determination of Antibacterial Activity—Agar Diffusion Plate Test (ISO 20645:2004)

**Figure 6:**
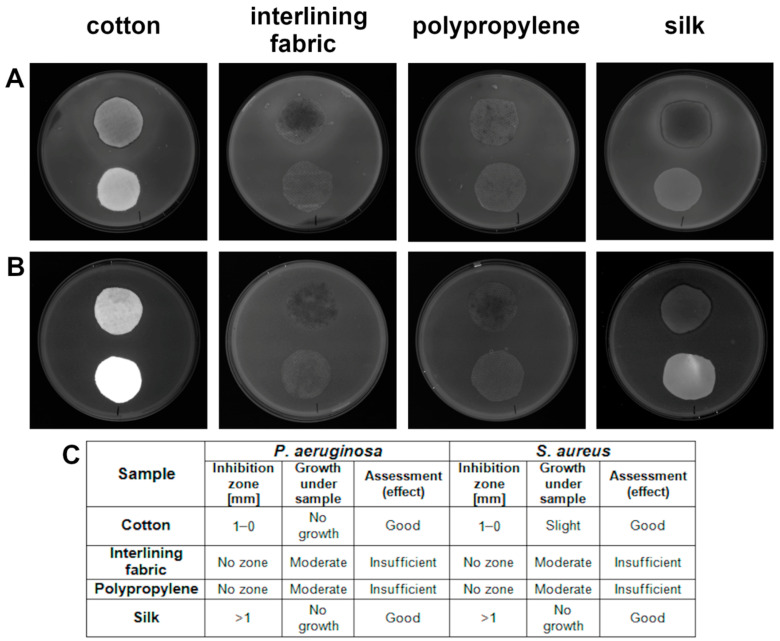
Antibacterial activity of textile materials against: (**A**) *P. aeruginosa*; (**B**) *S. aureus*; (**C**) evaluation according to ISO 20645:2004. Within each plate shown, implants placed closer to the upper edge of each plate are treated with nanomaterials and implants placed closer to the lower edge of each plate are control textiles.

According to ISO standard [22], a zone of inhibition in the range of 0–1 mm and more than 1 mm is assumed to be a good antibacterial effect of the materials tested. Such an effect was achieved in both bacteria strains in the cotton and silk groups (Figure 6). The results showed that polypropylene and interlining fabric had no antibacterial effect for *P. aeruginosa* and *S. aureus*.

### 3.8. Chorioallantoic Membrane (CAM) Assay

**Figure 7:**
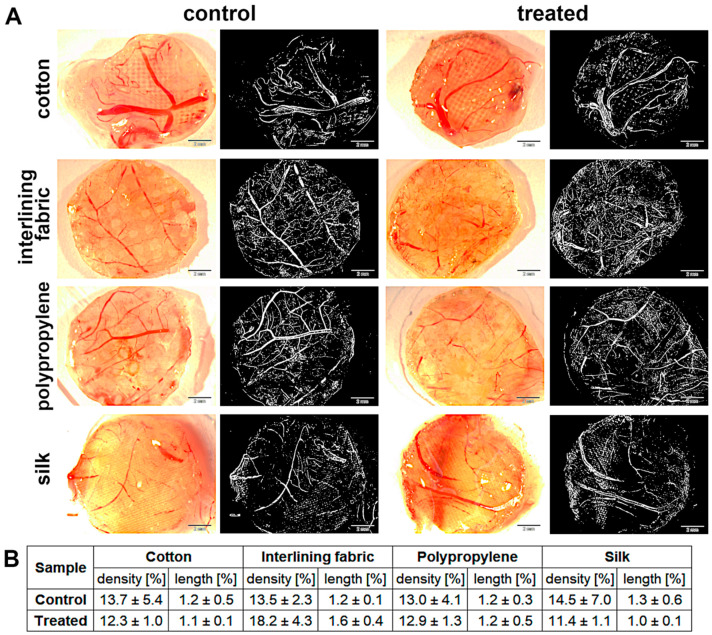
Angiogenesis analysis measured using CAM model: (**A**) measurements under stereomicroscope and the same measurements as binary images (sample pictures), respectively; (**B**) percentage of density and length of blood vessels. There were no significant differences between groups (*p*-value ≤ 0.05).

Angiogenesis was measured using the chicken embryo CAM implantation method. Both length and density in the nanoparticle treatment group were similar to the control (Figure 7). The biggest changes in the development of blood vessels in relation to the control were observed in the interlining fabric implant; however, the differences were insignificant.

## 4. Discussion

Currently, with traditional antibiotics being overused, the discovery of new antibacterial therapies appears to be essential for the further curing of infections. The possible solutions are currently attributed to nanotechnology, in which nanoparticles are considered to be therapeutic agents against the antibiotic resistance era [23]. The aim of our study was to evaluate an effective nanocomposite that is able to combat pathogenic bacteria. In order to achieve the intended effect, we conducted a two-level study in which the antibacterial effect was determined by the use of a nanocomposite as the main agent; subsequently, its application to textiles was defined.

In the present study, we demonstrated that AgNPs and GO flakes are able to create a complex structure with changed size distribution and zeta potential in comparison to individual nanoparticles (Figure 1). Silver nanoparticles, which have been used as medical agents since ancient times, are known for their tendency to create agglomerates which may weaken their antibacterial properties [24]. However, the formation of complexes composed of AgNPs and GO ensures better stabilization of the components [25]. This was evident in our study, in which the GO-Ag complex had zeta potential closest to ±30 mV, which indicated that the complex was characterized by better colloidal stability than the components alone. The size distribution of GO indicated the presence of large nanoparticles/agglomerates exceeding 1000 nm in diameter. However, as shown in the TEM analysis, GO was in the form of flakes, not a sphere, and therefore the definition of diameter is not completely clear. Nevertheless, the TEM visualization showed that GO still provides a large platform for attaching AgNPs to its surface, creating a carrier to transport smaller particles. It has been previously demonstrated that GO may be used as a carrier for other antibacterial components, including AgNPs [16,26], CuO [27], or Cu_2_O [28]. Moreover, Vi et al. [29] indicated the strong synergetic effect of GO-Ag due to its promotion of AgNPs on the scaffold formed of GO, so that AgNPs, well known for their antibacterial properties, might directly attack bacteria. The lack of colloidal stability of AgNPs was also visible in the hydrodynamic diameter exceeding 200 nm, as evidenced by the creation of agglomerates. Although it is recognized that smaller particles are more toxic [30], it may be slightly different when using composites. Results obtained from the research of Truong et al. [31] indicated that smaller GO-Ag did not have a better antibacterial effect because they only disturbed bacteria cells, while the bigger ones physically separated those cells from the nutrient medium and allowed silver ions to penetrate the cells’ structure, giving better antibacterial properties.

The antibacterial properties of nanomaterials are shown in Figure 3, where the inhibition of growth is visible by the zone around each well. The biggest zones of inhibition were observed in the bacteria in the wells with AgNPs as well as with the GO-Ag nanocomplex. GO had no antibacterial effect, giving the zone of inhibition growth was only 1 mm more than the well’s diameter itself. Similar results were obtained in previous studies where the zone of inhibition created by a complex of reduced GO (rGO) and AgNPs was twice the size of the rGO used on its own [32]. The diffusion of nanoparticles through the agar is hampered, but due to the highly satisfactory results obtained from agar diffusion tests, it appears to release silver ions from AgNPs as the main antibacterial agent [33]. This confirms our results where the zone of inhibition was similar in two groups: the bare AgNPs and the GO-Ag complex where AgNPs were used in the same concentration as in the individual AgNPs group. Considering that the GO nanoparticles were large flakes, the mechanism of releasing Ag ions from nanoparticles, which inhibits bacterial growth in GO-Ag nanocomposites, seems likely due to satisfactory antibacterial results from the agar well diffusion test, where bare GO did not cause an antibacterial effect and, due to its large size, may have diffusion limitations in agar.

The visualization of the interaction between nanomaterials and bacteria cells was analyzed by TEM (Figure 4). The main visible point of action was the accumulation around the bacteria cells, as well as on their surface, especially in the case of GO-Ag, where large GO flakes with smaller AgNPs coated the bacteria surface. The accumulation of the nanocomposite around the bacteria cells was confirmed by Moraes et al. [34]. The results clearly showed that nanomaterials interact with the cell membrane. This was also confirmed in our previous study [16], where the disruption of the cell membrane was expressed by LDH leakage in all groups of microorganisms after exposure to AgNPs and GO-Ag composites. However, due to the preparation of samples in which the cells were in one piece, it is possible that there are more interaction points between nanoparticles and bacteria than just adhesion to the membrane surface. As outlined previously, the exposure of NPs to bacteria cells involves action at many different levels, including disruption of the cell wall and membrane, generation of ROS and growth inhibition [16,35]. To determine the changes that occur in the bacteria metabolism, we performed the tetrazolium salt assay XTT. The results clearly showed that the cells’ metabolism was limited after exposure to the nanomaterials used in a dose-dependent manner (Figure 5A). Viability did not differ substantially between two bacteria species in the same groups; however, *P. aeruginosa* seemed to be more sensitive to GO-Ag, although the difference was marginal.

The higher susceptibility of *P. aeruginosa* was not visible in other analyses, for example, the agar well diffusion method, where *S. aureus* was more sensitive. The complexity of using a composite, however, leads to different parts interacting with different bacteria properties. On the one hand, the thin cell wall of Gram-negative bacteria does not provide protection against silver ions, which can penetrate the wall regardless of the presence of an outer layer [36]. On the other hand, it was reported that Gram-negative bacteria repel negatively-charged GO sheets, thereby indicating a higher viability than Gram-positive bacteria. GO sheets, despite their negative zeta potential, may interact with the negative surface charge of Gram-positive bacteria, even if, theoretically, these two charges should repel each other. The interaction may occur due to the stronger covalent bonds among carboxylic groups of GO and amines of peptidoglycan and amino acids [37]. In the FT-IR analysis (Figure 2), C-O and C-OH hydroxyl groups were found; thus, the hypothesis about their interaction with peptidoglycan seems to be probable.

The interaction of AgNPs with bacteria is defined as bimodal; the mechanism primarily consists of the impact on the cell membrane and wall, but subsequently leads to adsorption of NPs into the cell. ROS are also generated, which, despite their action inside the cell, are considered to be possible elements of the primary mechanism, as they counteract the antioxidant defense and lead to cell damage [38]. In our study, the highest level of ROS was in the nanocomplex groups; however, while bare AgNPs achieved a similar level, GO caused no oxidative stress, with the value close to the control groups (Figure 5B). A higher ROS production caused by GO-Ag than the level triggered by GO was also confirmed by Truong et al. [31].

**Figure 8:**
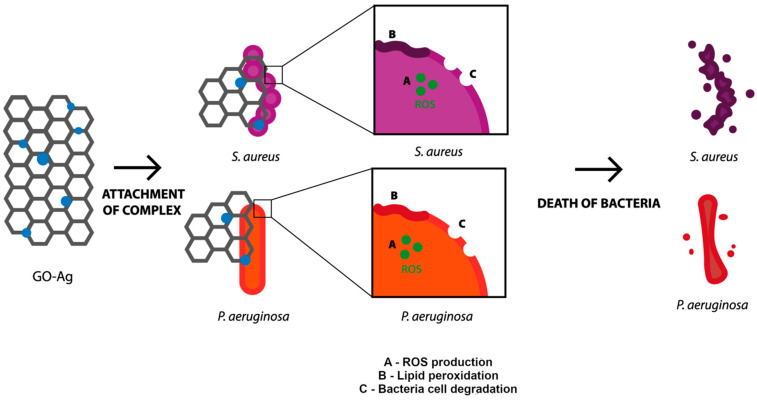
The mechanism of action of GO-Ag nanocomposite toward bacterial cells, including attachment of the complex, generation of ROS, lipid peroxidation and degradation of outer layer of bacteria resulting in bacteria death.

Exceeding the optimum level of ROS results in a lack of scavenging by antioxidants which are able to neutralize ROS. Consequently, the induced oxidative stress is high enough to start destroying cells by acting directly on membrane lipids [39]. In the present study, both AgNPs and the complex GO-Ag caused oxidative stress, as reflected in the increase in the lipid peroxidation level. Such a correlation was confirmed by Song et al. [40], who suggested that the main mechanism of the antibacterial action of GO-Ag is synergetic between cell membrane disruption and lipid peroxidation. The capability of ROS generation resulted in membrane lipid peroxidation [41]. The interaction between the levels of ROS and MDA was also determined in the case of bare AgNPs [42]. In our research, the concentration of MDA in both bacteria species was the highest after exposure to GO-Ag, compared with the control. Similarly high values were achieved in the group of AgNPs, but the concentration was lower than in the abovementioned example (Figure 5C). Curiously, the higher concentration of MDA and lower generation of ROS presented in *P. aeruginosa*, which is Gram-negative bacteria, indicated that they react in a different way than Gram-positive bacteria due to the presence of an outer membrane in the Gram-negative species. To summarize, the probable mechanism of action of the GO-Ag nanocomposite begins with the attachment to cells, which causes cell wall and membrane disturbance, but also the generation of ROS and lipid peroxidation, which, all together, ultimately lead to bacterial cell death (Figure 8).

Textile fabrics’ popularity is due to their wide range of applications, biodegradability, low cost and versatile usage. In order to create these materials with antibacterial properties, it is necessary to overcome some of their features, including hydrophilicity and porous structure, which make textiles a suitable growth medium for microorganisms [43]. Coating textiles in order to exhibit antibacterial features enables a wide range of usage, especially in the context of textiles from which healthcare products such as face masks are produced. It not only assures better properties, but also limits the spread of microorganisms, which are frequently drug-resistant. Farouk et al. [44] prepared nanocomposite-coated cotton and linen with highly antibacterial properties. In our study, only cotton and silk coated with the nanocomposite GO-Ag had antibacterial properties that had a good effect on both bacteria species, according to the standard ISO 20645:2004 for textile fabrics (Figure 6). A similar effect with the use of those two textiles was confirmed in other studies, where cotton coated with AgNPs had antibacterial properties demonstrated by the zone of inhibition towards the two bacteria *E. coli* and *S. aureus* [45]. Furthermore, silk coated with AgNPs inhibited the growth of bacteria strains even after 30 washes, implying a strong bond between the two structures [46]. GO contains many oxide functional groups by which it is able to bind, through the electrostatic interaction between two types of chemical groups, to textiles such as silk [47]. In our research, GO was used as a carrier for other nanoparticles; thus, it is probable that the nanocomposite attached to the silk through the interaction of the GO’s reactive chemical groups and the silk surface, which resulted in the best antibacterial properties of the tested textiles.

When using textiles or other kinds of materials, it is especially important to study their biocompatibility. An appropriate way to investigate such properties is the CAM assay [48], which is widely used as a toxicology model for different substances [49]. In recent years, this model has even become an intermediate step between in vitro and in vivo animal studies [50]. Conducting research in the field of nanomaterial toxicology is extremely challenging due to the large number of aspects that have to be taken into consideration, and the fact that many animals would be required to characterize a single substance [51]. In our study, the GO-Ag complex, as a factor influencing possible changes in the chorioallantoic membrane, did not cause the toxicity effect (Figure 7), as shown by the density and length of blood vessels from the CAM assay. The results were not diverse in relation to the non-treated control groups of the textile materials. The biggest differences were observed in the interlining group, but the difference was a few percentage points only. Similar results when using the nanoplatform GO-Ag were obtained by Wierzbicki et al. [52].

## 5. Conclusions

In conclusion, the effect of the application of GO decorated with silver nanoparticles towards two bacteria strains, *P. aeruginosa* and *S. aureus*, exhibited strong antibacterial potential (viability after the treatment of *P. aeruginosa* and *S. aureus* with the selected GO-Ag decreased to 27% and 31%, respectively, compared to AgNPs, when the viability of both species was 31% and 34%, accordingly). We demonstrated that, when bacteria cells are treated with a GO-Ag nanocomposite, more than the basic interaction point occurs. It was proven that the microbial cell membrane is disrupted, and oxidative stress and lipid peroxidation also contribute to cell death. Based on our results, nanocomposites of GO-Ag are able to overcome the limitation of single nanocomponents (zeta potential of GO-Ag was −23.6 ± 8.78 mV, while bare AgNPs was −18.7 ± 5.33 mV), and they may, therefore, constitute an effective agent towards resistant bacteria. Furthermore, nanocomposite-coated textiles showed excellent antibacterial properties, especially silk. Given the abovementioned facts concerning the effective action of nanocomposites and the coated textiles, we concluded that composites of GO-Ag may have practical applications in both coating textiles and using them independently, due to the efficient antimicrobial features that GO-Ag nanocomposites demonstrate.

## Data Availability

The data presented in this study are available on reasonable request from the corresponding author.
